# Shear behavior and construction method of steel shear keyed joints in precast segmental beams

**DOI:** 10.1038/s41598-023-37442-0

**Published:** 2023-07-10

**Authors:** Yu Zou, Tianyu Xiang, Dong Xu

**Affiliations:** 1 School of Architecture and Civil Engineering, Xihua University, Chengdu, 610039 China; 2 College of Civil Engineering, Tongji University, Shanghai, 200092 China

**Keywords:** Civil engineering, Mechanical engineering

## Abstract

The joints between segments represent weak points and introduce discontinuity into structures, therefore they are particularly significant in precast concrete segmental bridges. In this study, a new steel shear key was designed, and 6 full-scale tests were conducted. Various shear keys and joint types were taken as experimental parameters to study crack propagation, failure mode, shear slip, ultimate bearing capacity, and the residual bearing capacity of various joints under direct shear loading. The results show that the stiffness and shear capacity of steel shear keyed joints were higher than concrete key joints, and the structural system was more stable than concrete keyed joints at the moment of cracking. Both the concrete key and steel key epoxied joints suffered direct shear failure. However, different to the concrete epoxied joints which experienced brittle failure, steel key epoxied joints demonstrated a large residual capacity. Based on traditional segmental bridges construction, construction methods involving steel shear keyed joints included short-line match, long-line match, and modular methods are introduced. Finally, the feasibility of steel shear keyed joints construction was verified via engineering tests.

## Introduction

Precast concrete segmental bridge (PCSB) has developed rapidly because of its advantages of economical and safe design, fast and versatile construction, no disruption at ground level, excellent serviceability, low life-cycle costs, and quality control readily achieved^1–3^. Joints are the characteristics of the PCSB, which transmit the compressive and shear stress of joints^4,5^. But the reinforcement and concrete are discontinuous at the joint, which is the weak part of the structure^6,7^. Therefore, the shear performance of the joints has been attracting attention from researchers.

Jones et al.^8^ and Buyukozturk et al.^9^ concluded that the shear behavior of the dry keyed joints depends on the level of confining stress. Zhou et al.^10^ performed tests on shear transfer mechanisms of a series of full-scale keyed joints, and concluded that the epoxied joints have consistently higher shear strength than dry joints. Sangkhon et al.^11^ conducted an experimental study on the shape of keys and believes that the shear bearing capacity of semicircular and triangular keys is obviously better than that of trapezoidal keys, but semicircular and triangular keys are more prone to brittle failure. Yuan et al.^12^ found that the ductility of reinforced keys is superior to the plain concrete keys, and the internal tendon helps to improve the shear capacity of concrete keys. Choi et al.^13^ believe that the appropriate level of confining stress and epoxy can prevent sudden failure of joints at high amplitude cyclical loading conditions. Al-Rousan et al.^14^ study the influence of confining stress and concrete compressive strength on the shear behavior of dry single-keyed joints through non-linear FEA simulation, and a shear capacity formula was proposed. Zhang et al.^15^ performed full-scale tests on the shear behavior of multiple-keyed epoxy joints, and proposed a new formula considering the uneven distribution of shear stress. Alcalde’s^16^ test results show that the average shear stress transferred across the joint decreases with the number of keys but this influence is less appreciated as the confining stress increases. Zhan et al.^1^ conducted experimental investigations on the shear performance of key tooth joints (KTJ) under repeated loading, and found that compared to monotonic loading, the load-bearing capacity and stiffness of KTJ are significantly reduced under repeated loading due to the damage accumulation in concrete. Meanwhile, Zhan et al.^17^ indicated that the plain concrete keyed joint appears “direct shear” failure under direct loading, and adding internal steel bars will contribute to strengthening the connection of keyed joints and converting the dominant failure mode to the “crushing” mode. Luo et al.^18^ proposed analytical expression to predict the dynamic shear capacity of epoxied joints under different strain rates. Freitas et al.^19^ developed the failure envelopes considering the key multiaxial shear capacity with confinement pressure, P, moment, M, and torsion, T. Found that the ratios between moment and shear, M/V, and torsion and shear, T/V, control the failure mechanism. Smittakorn et al.^20^ and Beattie et al.^21^ carried out tests to assess the shear strength and deformation of joints utilizing steel fiber reinforced concrete(SFRC), and indicated that the addition of steel fibers improves the shear capacity and ductility of keyed joints. Jiang et al.^7^ found that the strength of SFRC keyed dry joints is 25% higher than that of conventional concrete keyed joints. Park et al.^22^ proposed an analytical model to predict the shear capacity of the SFRC keyed joints through experimental investigation. Voo et al.^23^ , Gopal et al. ^24^ and Kim et al.^25^ performed tests on the shear capacity of the UHPC keyed joint and found that the failure loads increased with the number of shear keys. Hu et al.^4^ obtained a shear strength prediction method of the UHPC keyed dry joint based on previous experimental results and numerical simulation. Sun et al. ^26^ found that the ductility of the shear keys of ordinary steel bars is better, while the shear keys of FRP bars show greater brittleness. Issa et al.^27^ found that the AASHTO specification neglects the contribution of epoxy resins and underestimates the shear strength of the single key epoxied joint. Rombach et al.^28^ proposed a new design model for dry joints, and believe that the AASHTO specification overestimates the bearing capacity of dry joints. Turmo et al.^29^ thought that the safety factor (0.75) should be considered in the calculation of the shear strength of concrete keyed joints by the AASHTO formula. Shamass et al.^30^ recommend reducing the friction coefficient used in the AASHTO code equation when high confining pressure is applied.

To simplify joint type, improve joint shear force transmission, and enhance joint bearing capacity and ductility, a steel shear key was designed for precast segmental bridges. To fully study the mechanical properties of the steel shear key, six samples were designed, and the shear key type and joint types were used as experimental parameters. Taking crack propagation, failure mode, ultimate bearing capacity, residual bearing capacity, and shear slip as research focuses, the mechanical properties of steel keys and normal concrete keys were compared. Steel shear keyed joint construction methods were designed and engineering tests were conducted based on short-line and long-line match methods. Finally, it is proposed that steel shear keys have wide applicability from the viewpoint of reliable force, simple and convenient construction.

## Experimental program

### Steel shear key

**Figure 1:**
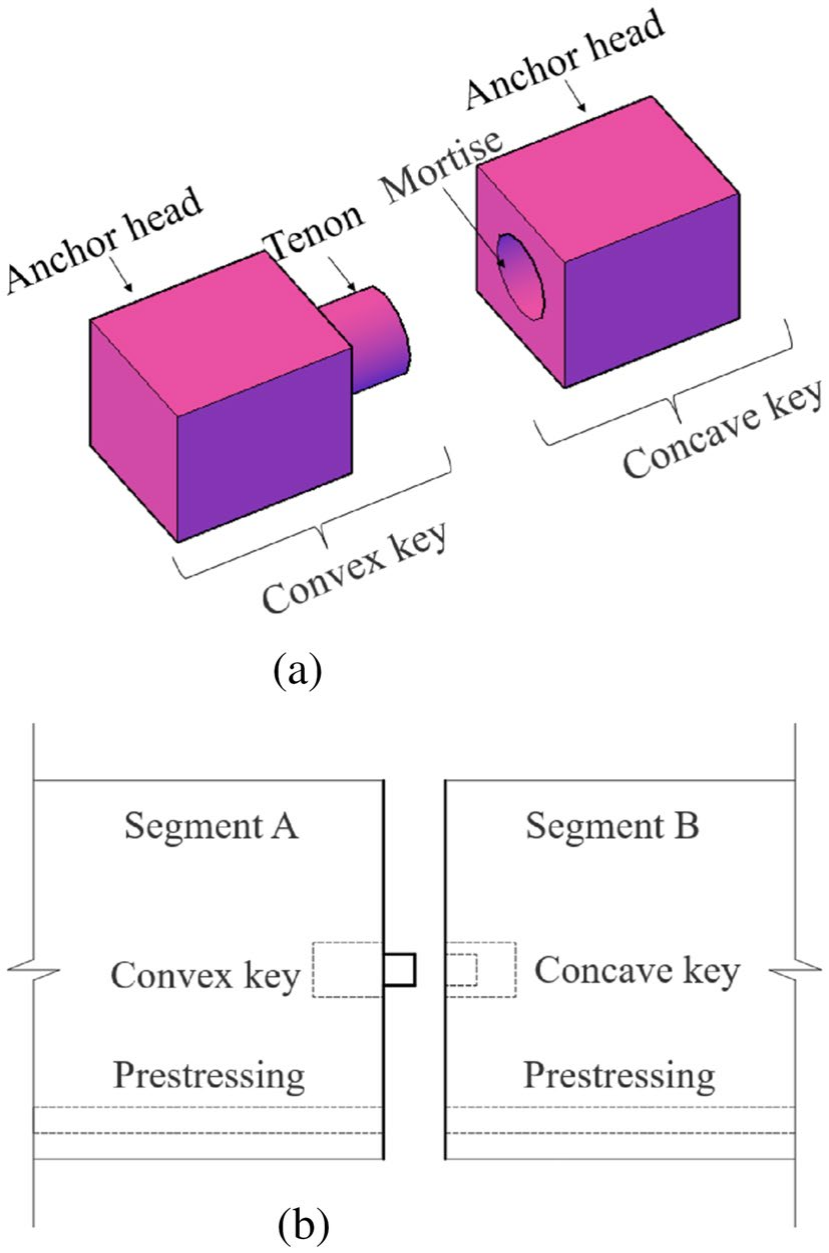
Steel shear key schematic diagram^31^ (**a**) component (**b**) steel shear key joint.

The steel shear key sets are shown in Fig. 1a, including a convex key and a concave key. The concave-convex keys are connected by a mortise and a tenon, and the anchor heads are embedded separately in the segments. In segmental assembly, under the action of longitudinal prestressing force (internal or external), the joints are assembled and connected by matching concave and convex keys, as shown in Fig. 1b.

### Equivalent shear key design

The concrete keys(CK) were made of C50 commercial concrete^32^ and were designed according to AASHTO code^33^. The steel shear keys (SSK) were finished with Q235^32^, and the finish machining had a manufacturing error 0.001 mm. To facilitate matching construction, the tenon and mortise were designed with a construction gap of 0.2 mm, and a 45° slope foot was designed in the tenons.

Roberts and Breen^34^ believed that the bearing capacity of the keyed tooth joints is provided by the shear keys and the interfacial friction, and proposed that the shear resistance Eqs. (1) be written into AASHTO^33^.

1 $$ V_{j} = A_{k} \sqrt {f_{c}^{^{\prime}} } \left( {0.996 + 0.205\sigma_{n} } \right) + 0.6A_{sm} \sigma_{n} $$

In Eqs. (1), the shear resistance of the shear key is

2 $$ V_{k} = A_{k} \sqrt {f_{c}^{^{\prime}} } \left( {0.996 + 0.205\sigma_{n} } \right) $$

The practical calculation formula of shear strength is

3 $$ F = \tau \cdot A $$

where $V_{j}$ is the shear capacity of joints, N; $V_{k}$ is the shear capacity of keys, N; $A_{k}$ is the total area at the root of keys, mm^2^; $f_{c}^{^{\prime}}$ is compressive strength of concrete cylinder, MPa; $\sigma_{n}$ is the confining stress, MPa; $A_{sm}$ is the area of non-keyed , mm^2^; $\tau$ is the shear stress, MPa; $A$ is the shear area, mm^2^; $F$ is the shear force, N.

**Figure 2:**
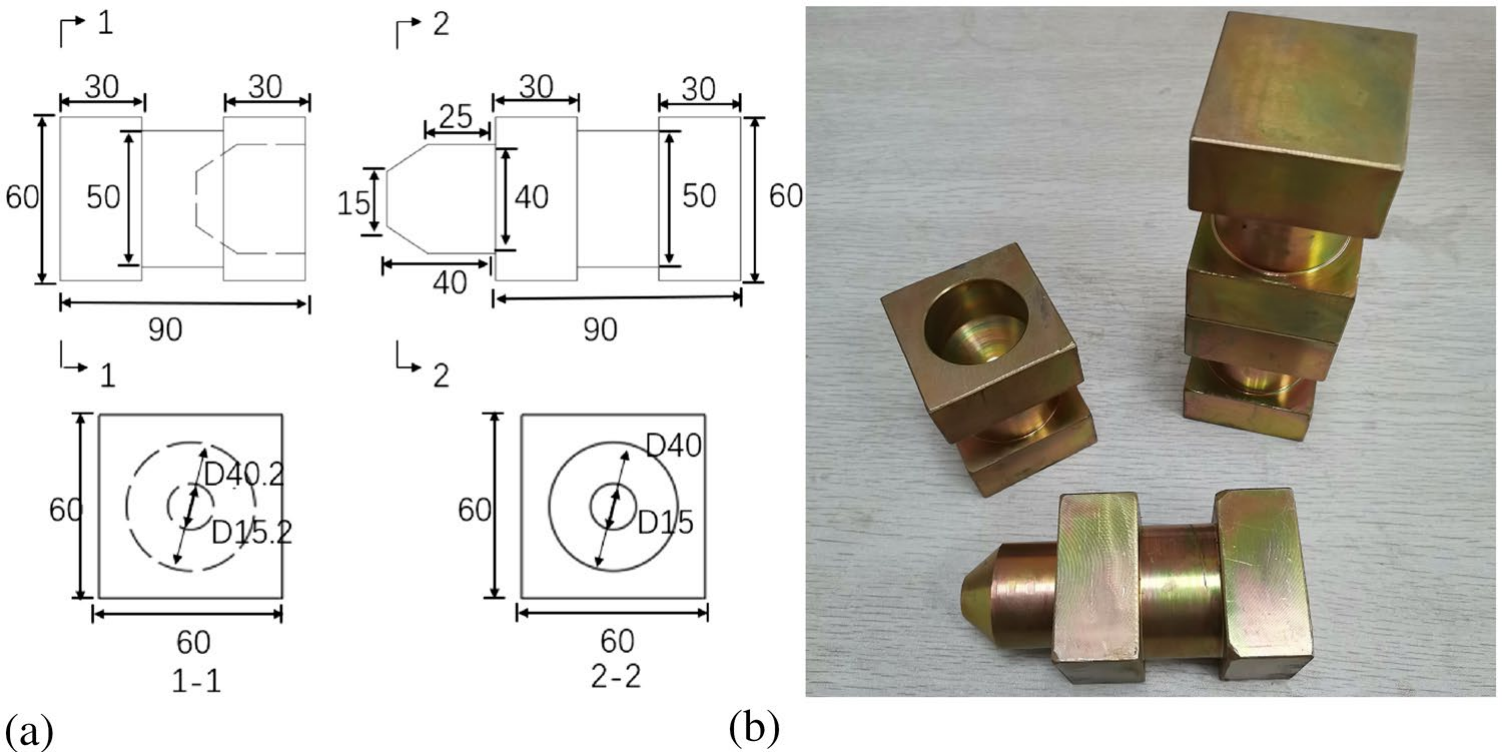
Design of shear key^31^ (**a**) CK (**b**) SSK (unit: mm).

The shear key’s design was based on material shear strength and geometric size. Based on the equivalent of shear strength, Eqs. (2) and (3) were used to design the dimensions of the concrete and steel keys. The geometric dimensions of CK and SSK are shown in Fig. 2a and b.

### Test specimens design

**Figure 3:**
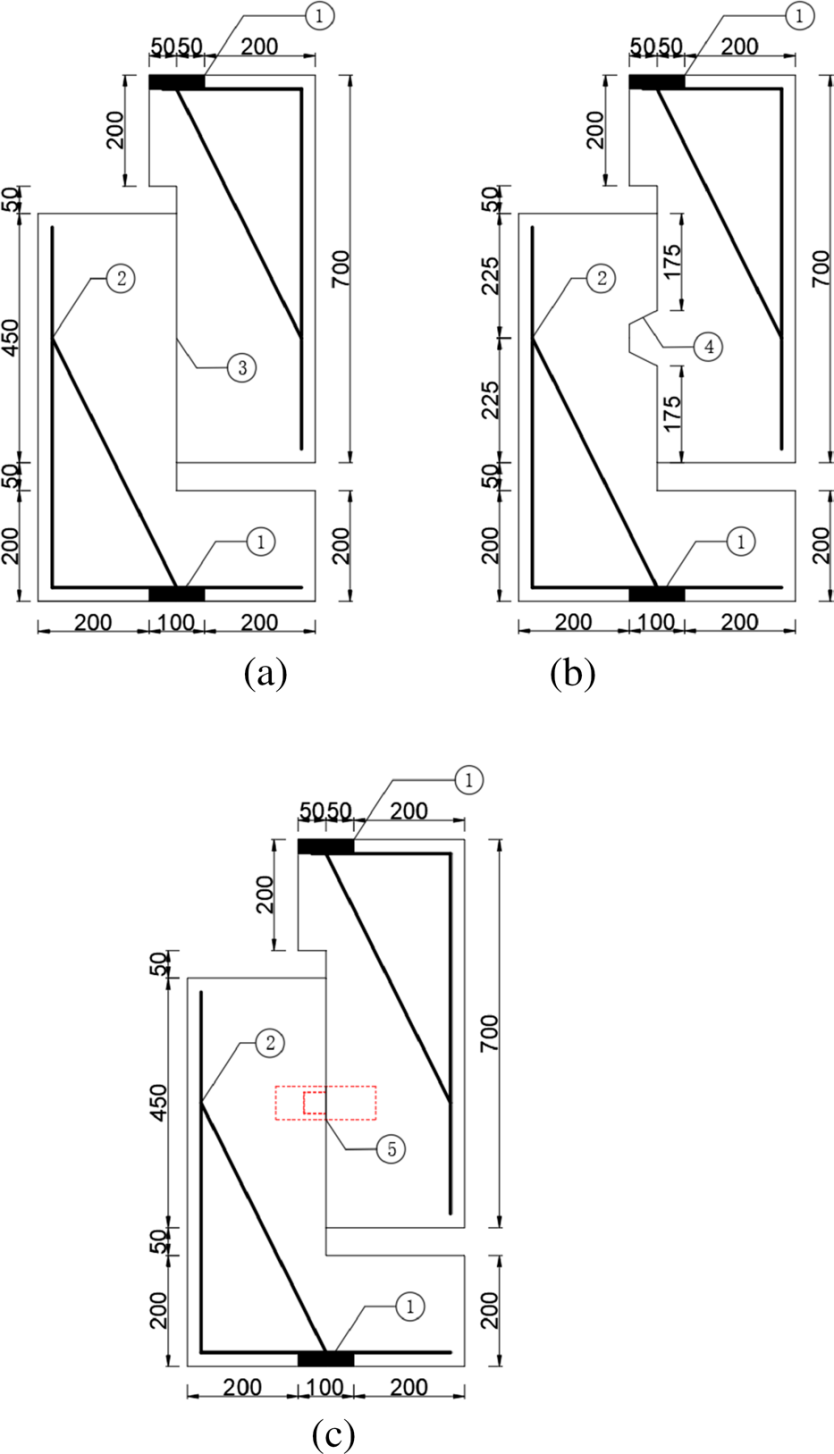
Geometric dimensions of specimens^31^ (**a**) flat joint (**b**) concrete key joint (**c**) steel key joint. 1. Embedded steel plates (Q235^32^, 200 mm × 100 mm × 25 mm. 2. Constructional reinforcement (HRB400^32^, 16 mm). 3. Flat surface. 4. CK. 5. SSK (unit: mm).

The specimen's design referred to the experimental model of Yuan^36^. The shear slip gap of the convex and concave specimens was 50 mm, and the thickness of each test specimen was 200 mm. Figure 3a–c and Table 1 list the parameters of each test specimen.

**Table 1:** Identification of the specimens and parameters.

Specimen ID	Concrete strength	Joint type	Joint area/mm^2^	Confining stress/MPa	Key type	Key's number
DS1	C50	Dry	200 × 450	1	–	0
DS2	C50	Dry	200 × 450	1	CK	1
DS3	C50	Dry	200 × 450	1	SSK	1
DS4	C50	Epoxied	200 × 450	1	–	0
DS5	C50	Epoxied	200 × 450	1	CK	1
DS6	C50	Epoxied	200 × 450	1	SSK	1

### Test specimen preparation

**Figure 4:**
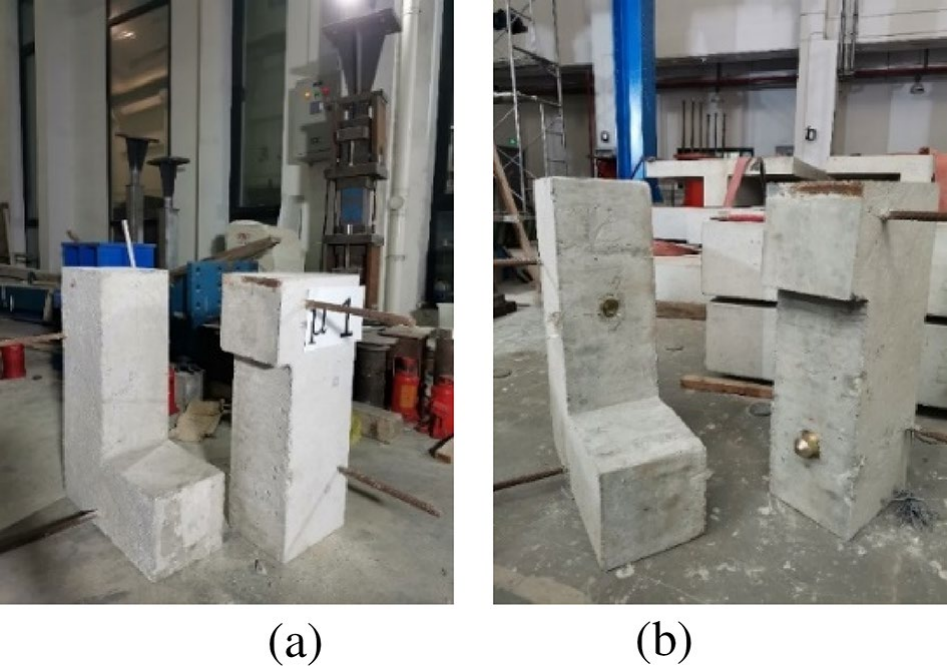
Test specimens (**a**) concrete joint (**b**) steel key joint.

Commercial concrete and commercial epoxy glue were used for specimen making. And the materials characteristic cited the test results of Zou^37^. The specimens used matching pouring, and the specimen models are shown in Fig. 4.

### Experiment setup and test procedure

**Figure 5:**
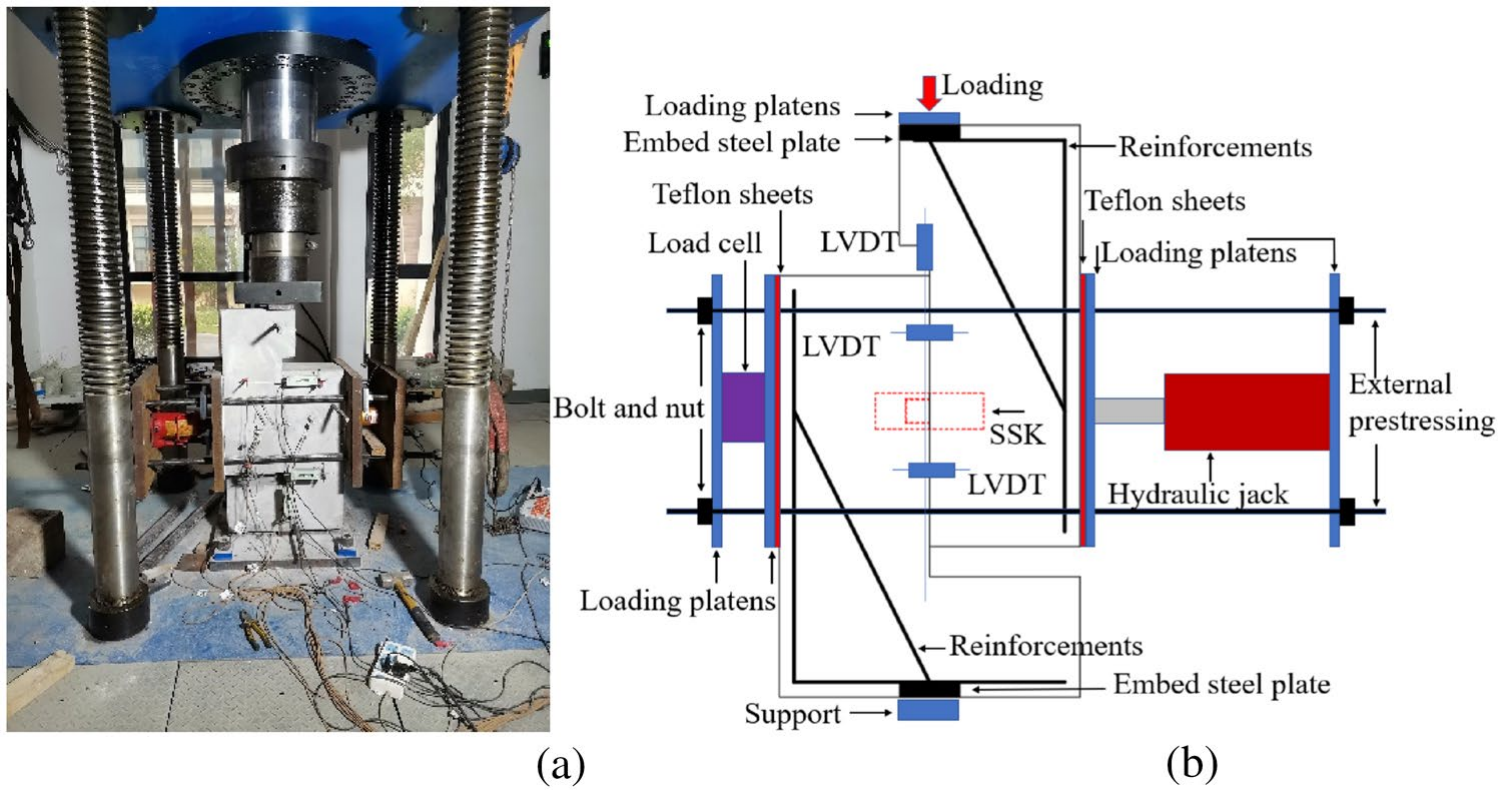
Test setup^31,35^ (**a**) Test machine (**b**) Loading system.

The test machine and test setup are shown in Fig. 5a and b. Displacement-control tests for all specimens were conducted at a constant stroke rate of 0.1 mm/min^7^. The confining stress, simulating the effect of prestressing in segmental bridges, on all specimens was 1 MPa. The shear slip between joints was measured by LVDTs, and the layout of the transducers is shown in Fig. 5b. The support, consisting of a rectangular steel plate, was braced against bending and distortion and restricted against horizontal movement^31^. The loading point, support point and joint were collinear.

## Experimental results and analysis

### Dry joints

#### Concrete joints

The experimental data is summarized in Table 2. The maximum bearing capacity of the flat joint was $F_{max} = 56.7{\text{kN}}$.

**Table 2:** Mechanical properties of dry joints.

SpecimensID	Cracking load ${\varvec{V}}_{{{\varvec{cr}}}} \left( {{\varvec{kN}}} \right)$	Ultimate load ${\varvec{V}}_{{\varvec{u}}} \left( {{\varvec{kN}}} \right)$	$\frac{{{\varvec{V}}_{{{\varvec{cr}}}} }}{{{\varvec{V}}_{{\varvec{u}}} }}\left( \user2{\% } \right)$	Confining stressin initial state ${\mathbf{P}}_{{{\varvec{in}}}} \left( {{\varvec{kN}}} \right)$	Confining stressin cracking state ${\mathbf{P}}_{{{\varvec{cr}}}} \left( {{\varvec{kN}}} \right)$	$\frac{{{\mathbf{P}}_{{{\varvec{cr}}}} - {\mathbf{P}}_{{{\varvec{in}}}} }}{{{\mathbf{P}}_{{{\varvec{in}}}} }}\left( \user2{\% } \right)$
DS1	-	56.7	-	90	-	-
DS2	174.8	181.6	96.3	90	120.0	33.3
DS3	271.2	314.1	86.3	90	97.6	8.4

**Figure 6:**
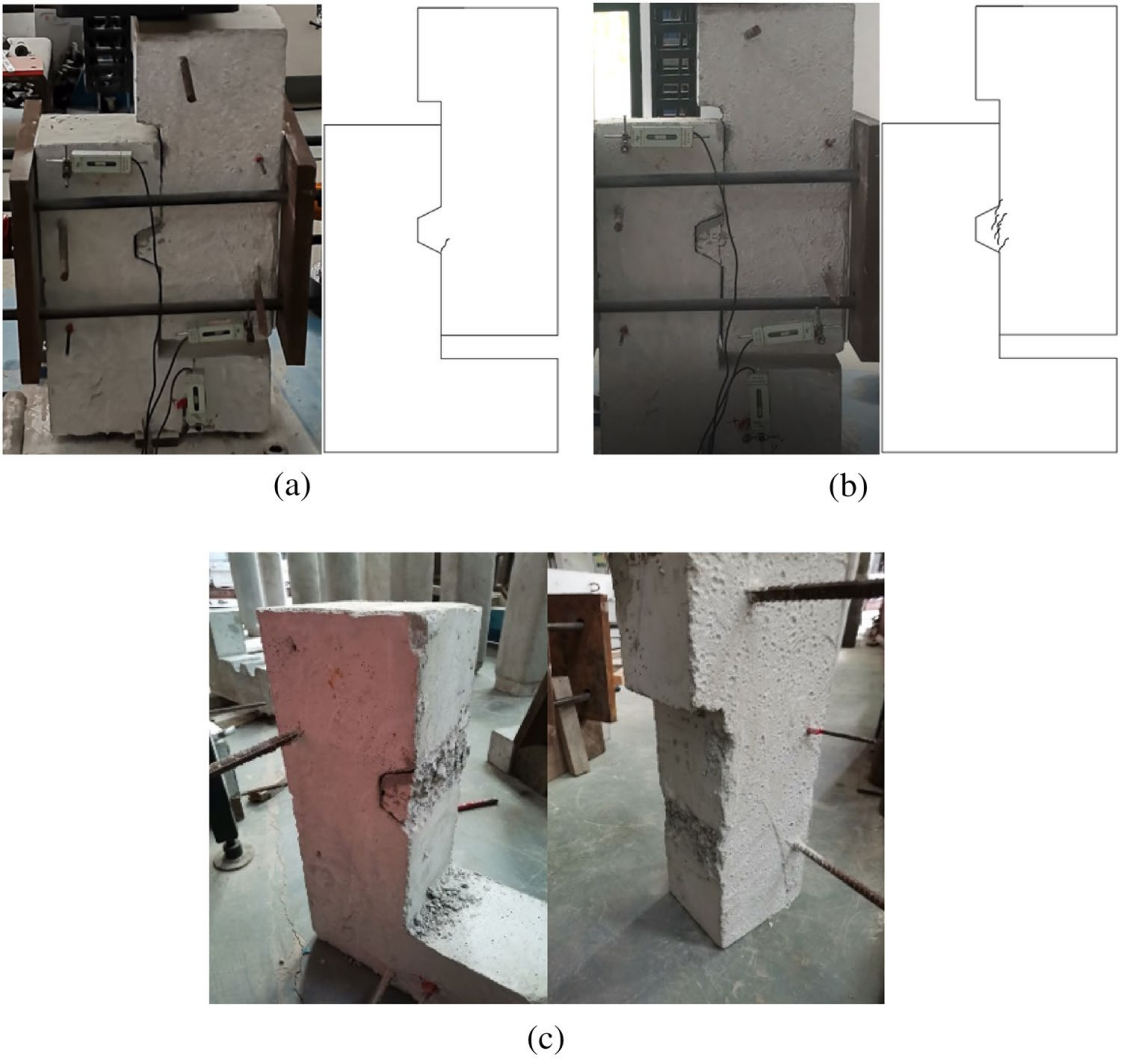
Crack formation and failure mode of DS2. (**a**) initial crack (**b**) crack propagation (**c**) failure mode.

The cracking load of the concrete key joint was 174.8kN, the ultimate load was 181.6kN, the ratio of cracking load to ultimate load was 96.3%, and the confining stress increased by 33.3% at the moment of cracking. The crack propagation and failure mode of the concrete key are shown in Fig. 6. The initial crack, which appeared at the lower edge of the convex key, was around 70° horizontally. Subsequently, cracks at the root of the convex key developed rapidly, and several small parallel cracks appeared, merging and forming a shear main crack with the initial crack.

From the experimental data and observable phenomena, it can be seen that the process from cracking to shear failure of the concrete key joints was rapid and brittle. The experimental results were consistent with those of Zhou^10^ and Yuan^36^ et al.

#### Steel shear keyed joint

**Figure 7:**
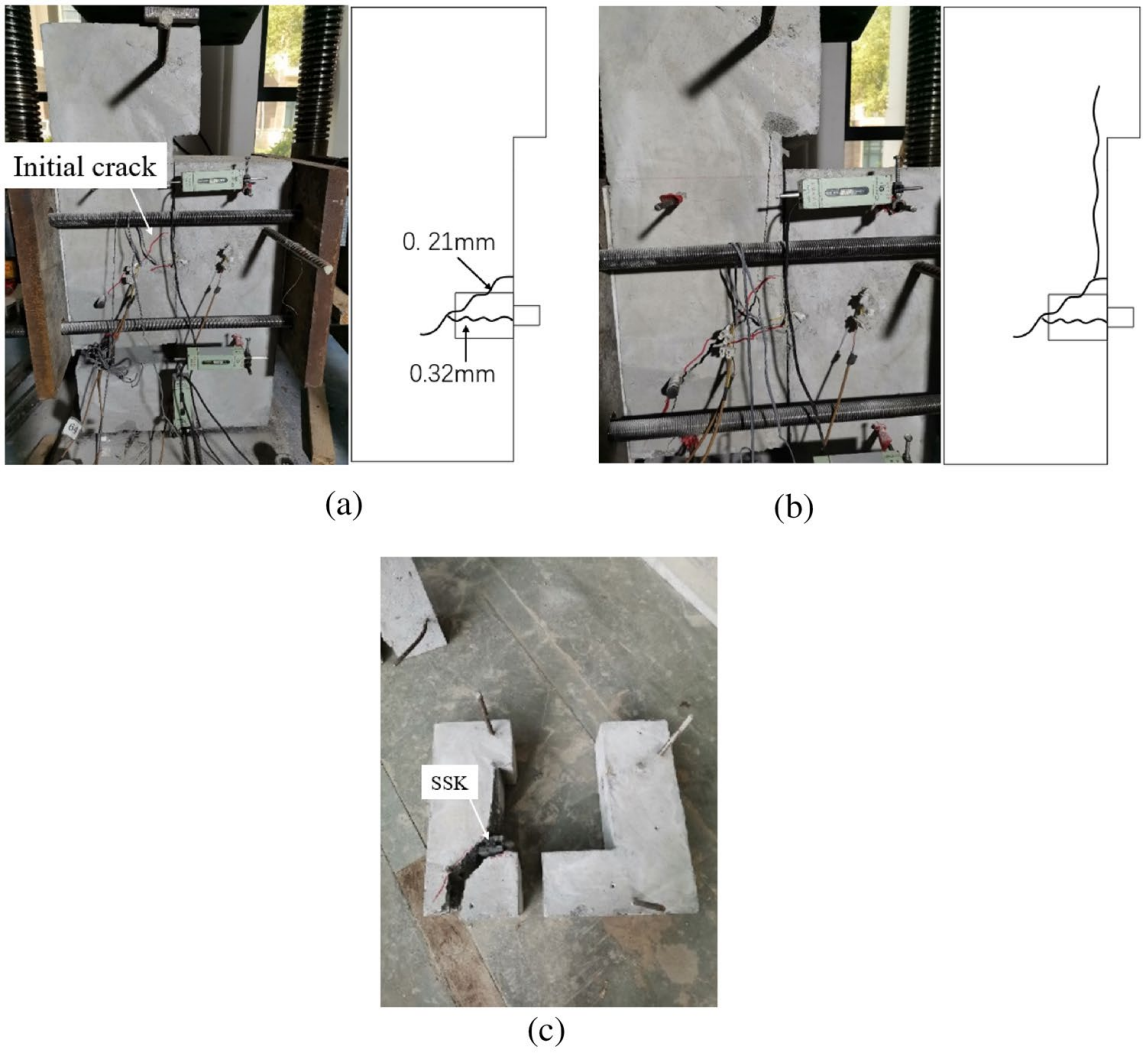
Crack formation and failure mode of DS3. (**a**) initial crack (**b**) crack propagation (**c**) failure mode.

The cracking load of the steel keyed joint was 271.2kN, the ultimate load was 314.1kN, the ratio of cracking load to ultimate load was 86.3%, and the confining stress increased by 8.4% at the moment of cracking. The crack propagation and failure mode of the steel key joint are shown in Fig. 7. Two initial cracks simultaneously appeared near the convex keys, one of which was close to the horizontal and the other was close to 45°, penetrating in the specimen thickness direction. When the cracks appeared, the stiffness of the specimens decreased suddenly and the loading force instantaneously decreased. As loading continued, no new cracks appeared, but the width and length of the existing cracks developed significantly. The horizontal and oblique cracks gradually formed a main crack near 45°, and gradually developed towards the loading point, without crossing the joint surface. Upon the specimen being damaged, the convex key concrete formed two completely separate bodies along the main crack, while the concave key concrete did not crack and break, and the steel key was not damaged.

### Epoxied joints

#### Concrete joints

**Figure 8:**
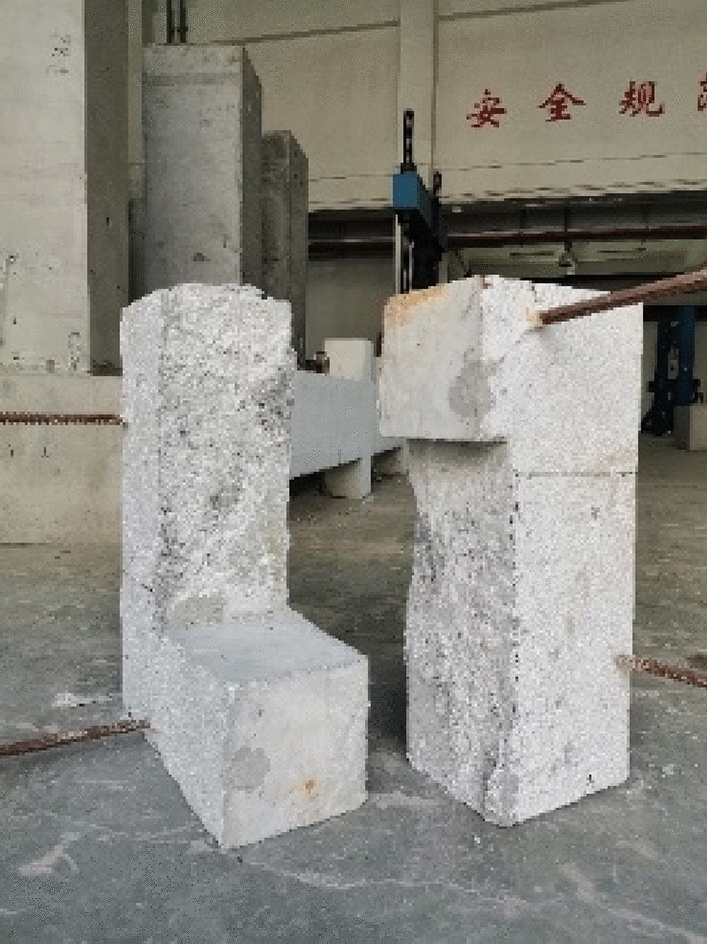
Failure mode of DS4.

Initially, the flat joint was very stiff, and the loading force increased rapidly. Until peak loading $F_{max} = 479.5{\text{kN}}$, the specimen occurred direct shear failure along the joint position, and the confining stress increased by 85.33%, as shown in Table 3. The failure mode is shown in Fig. 8.

**Table 3:** Mechanical properties of epoxied joints.

Specimens ID	Cracking load ${\varvec{V}}_{{{\varvec{cr}}}} \left( {{\varvec{kN}}} \right)$	Ultimate load ${\varvec{V}}_{{\varvec{u}}} \left( {{\varvec{kN}}} \right)$	$\frac{{{\varvec{V}}_{{{\varvec{cr}}}} }}{{{\varvec{V}}_{{\varvec{u}}} }}\left( \user2{\% } \right)$	Confining stressin initial state ${\mathbf{P}}_{{{\varvec{in}}}} \left( {{\varvec{kN}}} \right)$	Confining stress in cracking state ${\mathbf{P}}_{{{\varvec{cr}}}} \left( {{\varvec{kN}}} \right)$	$\frac{{{\mathbf{P}}_{{{\varvec{cr}}}} - {\mathbf{P}}_{{{\varvec{in}}}} }}{{{\mathbf{P}}_{{{\varvec{in}}}} }}\left( \user2{\% } \right)$
DS4	479.5	479.5	100	90	166.80	85.33
DS5	529.5	529.5	100	90	166.49	84.99
DS6	685.8	685.8	100	90	110.92	23.24

**Figure 9:**
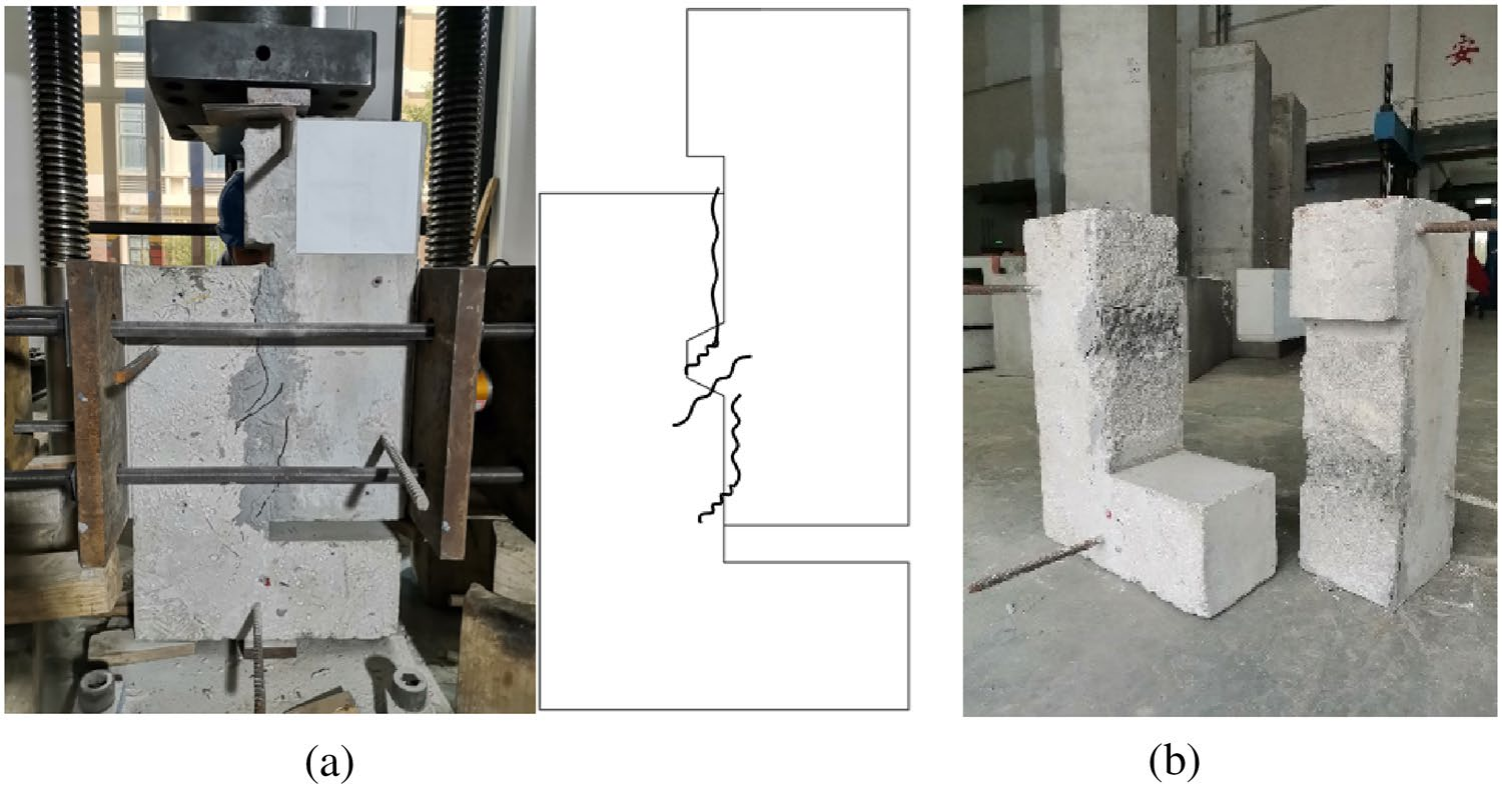
Crack formation and failure mode of DS5. (**a**) crack propagation (**b**) failure mode.

The crack propagation and failure mode of the concrete key joint are shown in Fig. 9. When the crack appeared, the loading force was 529.5kN and the confining stress increased by 84.99%. The cracks developed along the joint position and penetrated in the thickness direction. When the specimen was damaged, the cracks at the root of the convex key connected vertically to form a shear surface. The concrete mortar layer on the joint surface fell off, and the aggregate leaked out. However, the epoxy glue was not damaged. After the crack appeared, the stiffness of the specimen noticeably decreased, the loading force plummeted, and the cracking load was the ultimate load.

#### Steel shear keyed joint

**Figure 10:**
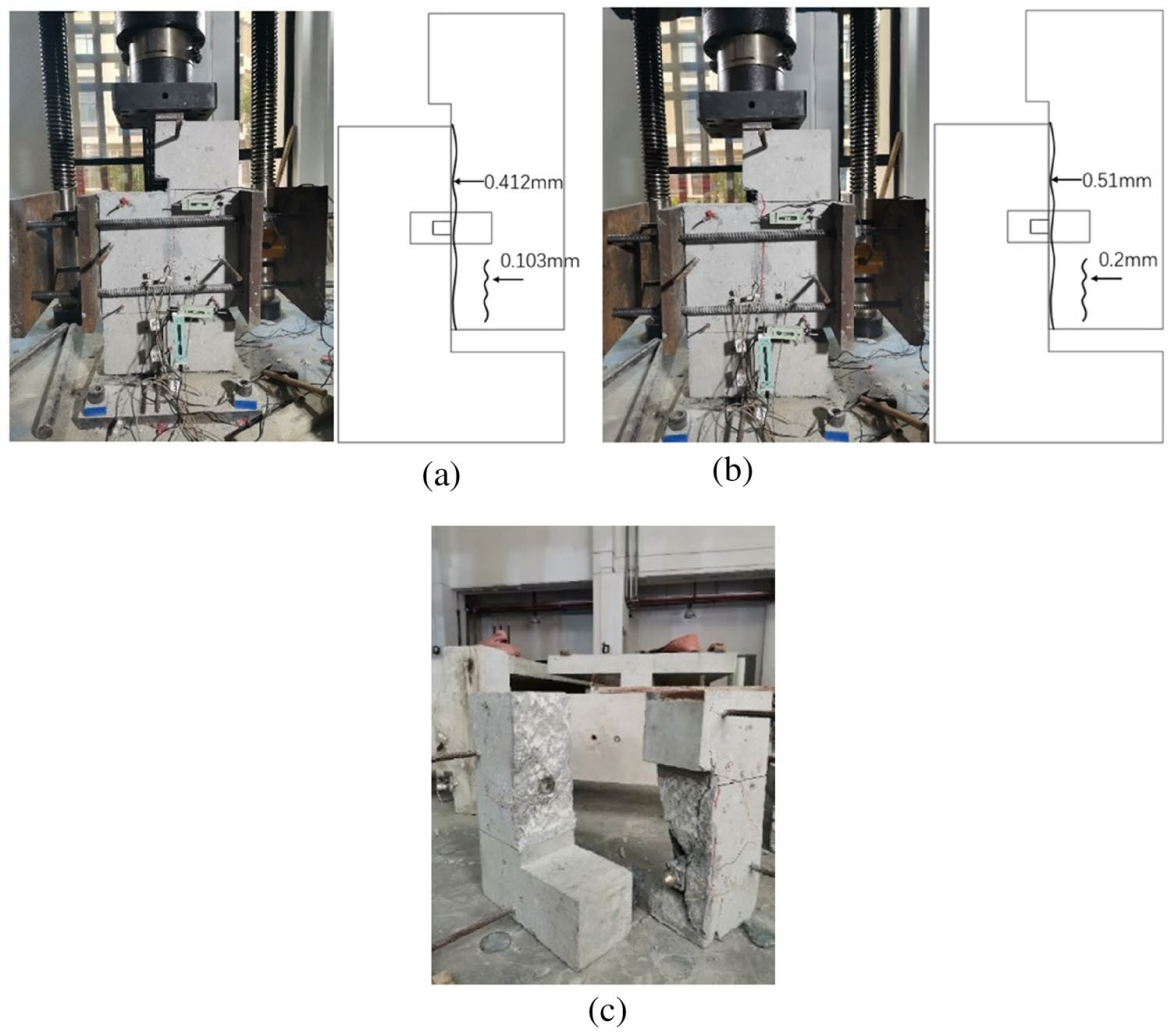
Crack formation and failure mode of DS6. (**a**) initial crack (**b**) crack propagation (**c**) failure mode.

Initially, the specimen was very stiff. When the loading force reached 685.8kN, the specimen experienced direct shear cracking along the joint position. The crack occurred in the concrete mortar layer, with a width greater than 0.2 mm, as shown in Fig. 10. After the cracks appeared, the confining stress increased by 23.24%. As opposed to the concrete key epoxied joint, the steel key epoxied joint quickly realized the redistribution of internal force after the occurrence of direct shear cracks, achieved a new mechanical balance, enabling it to still bear the load. In addition, the specimen stiffness visibly reduced, the loading force did not increase, no new cracks appeared in the specimen, the initial crack width increased, and the load–displacement curve entered the horizontal stage.

Following damage, both the concave and convex specimens were completely separated along the joint surface, the mortar layer on one side of the joint surface was completely stripped off to the other side of the specimen, the coarse aggregate was clearly visible on the joint surface, and the steel shear key did not suffer any deformation or damage.

## Shear mechanical properties of joints

### Influence of shear key types

**Figure 11:**
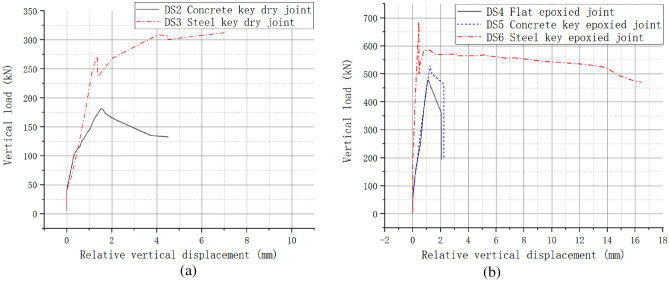
Effect of shear key types on mechanical properties of joints (**a**) dry joint (**b**) epoxied joint.

The load–displacement curves of dry joints are shown in Fig. 11a. The ultimate bearing capacity of the concrete key dry joint and steel key dry joint was 181.6kN and 272.8kN respectively, which was 2.20 and 3.81 times higher than that of the flat dry joint. The ultimate bearing capacity of the steel key dry joint was 72.96% higher than that of the concrete key dry joint. Direct shear failure occurred in the concrete key dry joints, and the specimens showed sudden shear slip. However, the concave and convex keys still maintained interlocking under confining stress, after the ultimate bearing capacity of the steel key dry joint was reached. The specimen could still bear the load and the load–displacement curve featured a long horizontal stage. The ratio of residual bearing capacity to ultimate bearing capacity was large.

The load–displacement curves of epoxied joints are shown in Fig. 11b. The ultimate bearing capacity of the concrete epoxied key joint and steel key epoxied joint were 529.5kN and 685.8kN respectively, which was 10.42% and 43.02% higher than that of the flat epoxied joint. The ultimate bearing capacity of the steel key epoxied joint was 29.52% higher than that of the concrete key epoxied joint. The steel key epoxied joint and concrete epoxied key joint all exhibited direct shear failure.

As shown in Table 4, the concrete joints (dry and epoxied) had a large shear slip after direct shear failure. However, the shear slip of the steel key joints was obviously smaller than that of the concrete key joint, as the shear stiffness of the steel key joint was sufficient.

**Table 4:** Shear slip at cracking.

Joint types	Dry joints		Epoxied joints	
Shear slip (mm)	Concrete key	Steel key	Concrete key	Steel key
Before cracking	1.63	1.35	1.24	0.44
After cracking	3.73	1.38	2.23	0.46
$\Delta$	2.10	0.03	0.99	0.02

### Influence of joint types

**Figure 12:**
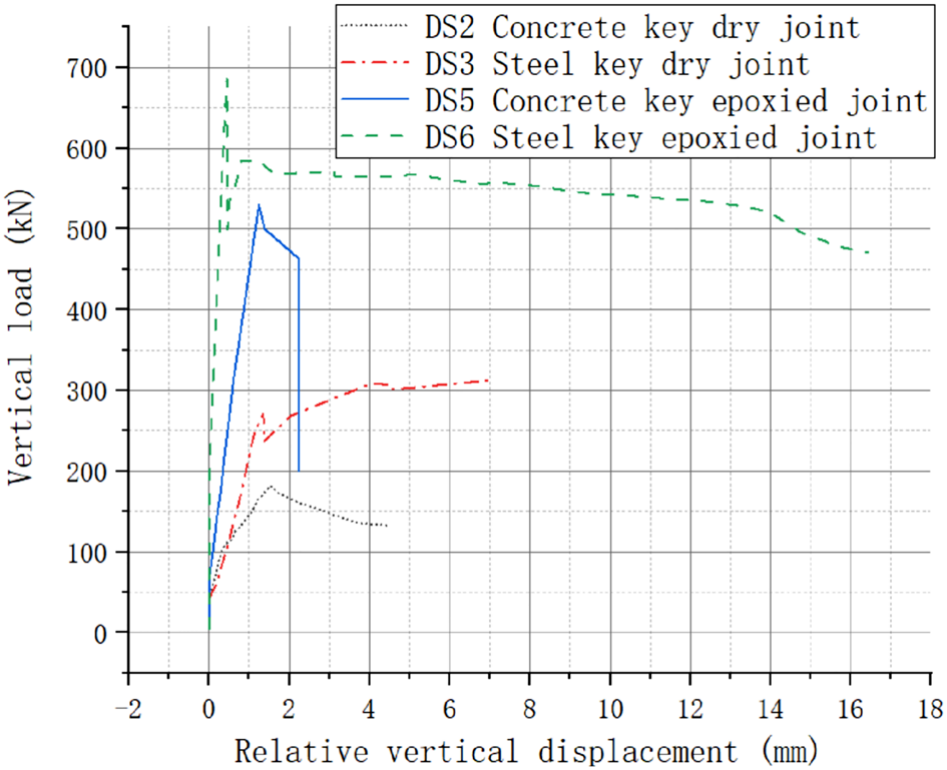
Effect of joint types on mechanical properties of joints.

The load–displacement curves are shown in Fig. 12. The stiffness of the concrete key epoxied joints was higher than the dry joints. Compared with dry joints, the ultimate bearing capacity of the concrete key epoxied joints increased by 191.57%. However, the concrete key epoxied joints showed more obvious brittle damage. Joint resistance was only provided by interfacial friction after the concrete key epoxied joint was damaged.

Compared with the dry joints, the ultimate bearing capacity of the steel shear keyed joint increased by 118.33%. With the peak loading force, the steel key specimen suffered direct shear cracking along the joint position. After cracking, the resistance of the epoxy glue failed, and consequently the stress state of the steel key epoxied joint was similar to that of the dry joint. The joint resistance was provided by the steel key and interface friction.

### The force transfer mechanism

**Figure 13:**
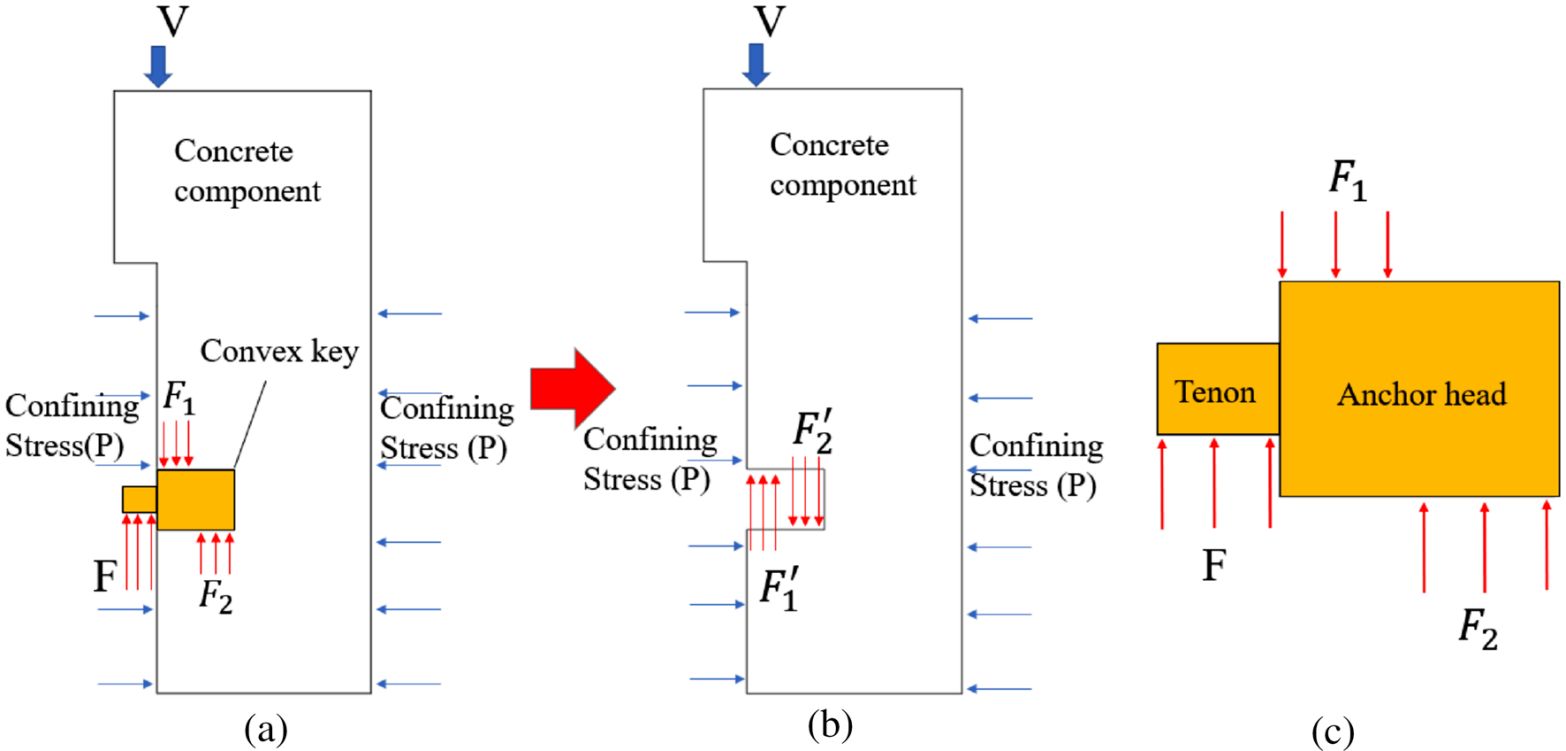
Simplified mechanical diagram (**a**) Convex key specimen (**b**) Concrete component (**c**) Steel key.

**Figure 14:**
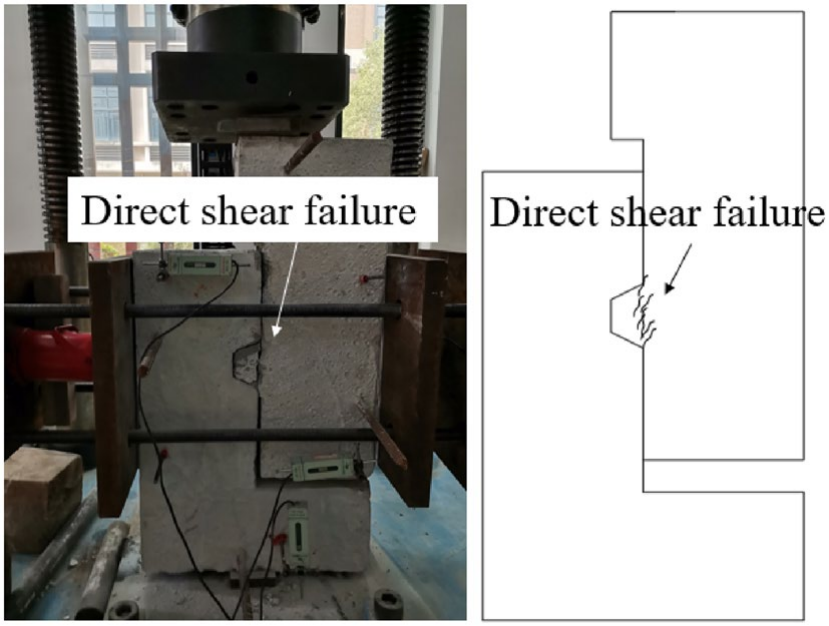
Concrete key failure mode.

In order to explore the force transfer mechanism and the shear behavior of steel keyed joints, the specimens were deconstructed and the detached body was analyzed, and the convex key also exhibited a leverage effect. Therefore, the convex key was selected as the research object for mechanical analysis of the detached body. The mechanical diagram of concrete component is shown in Fig. 13a and b, while the mechanical diagram of the convex key is shown in Fig. 13c. Based on the simplified diagram, the force transfer mechanism to obtain the steel tenon joint is as follows: Under the confining stress, the steel keyed joint relies on the contact pressure between the steel key and the concrete to transfer the shear force between the joint. On the other hand, the concrete keyed joints were damaged by direct shear failure. The shear plane was located at the root of the key, as show in Fig. 14.

## Construction method

For the construction of precast segmental concrete bridges, both short-line match and long-line match methods have been widely applied both at home and abroad. Based on existing established methods, short-line match and long-line match methods were applied to the construction design of steel shear keyed joints. At the same time, the modular construction method, with a higher degree of standardization, was designed by combining the advantages of simple fabrication and easy installation of the steel shear keys.

### Short-line match method

#### Construction method design

**Figure 15:**
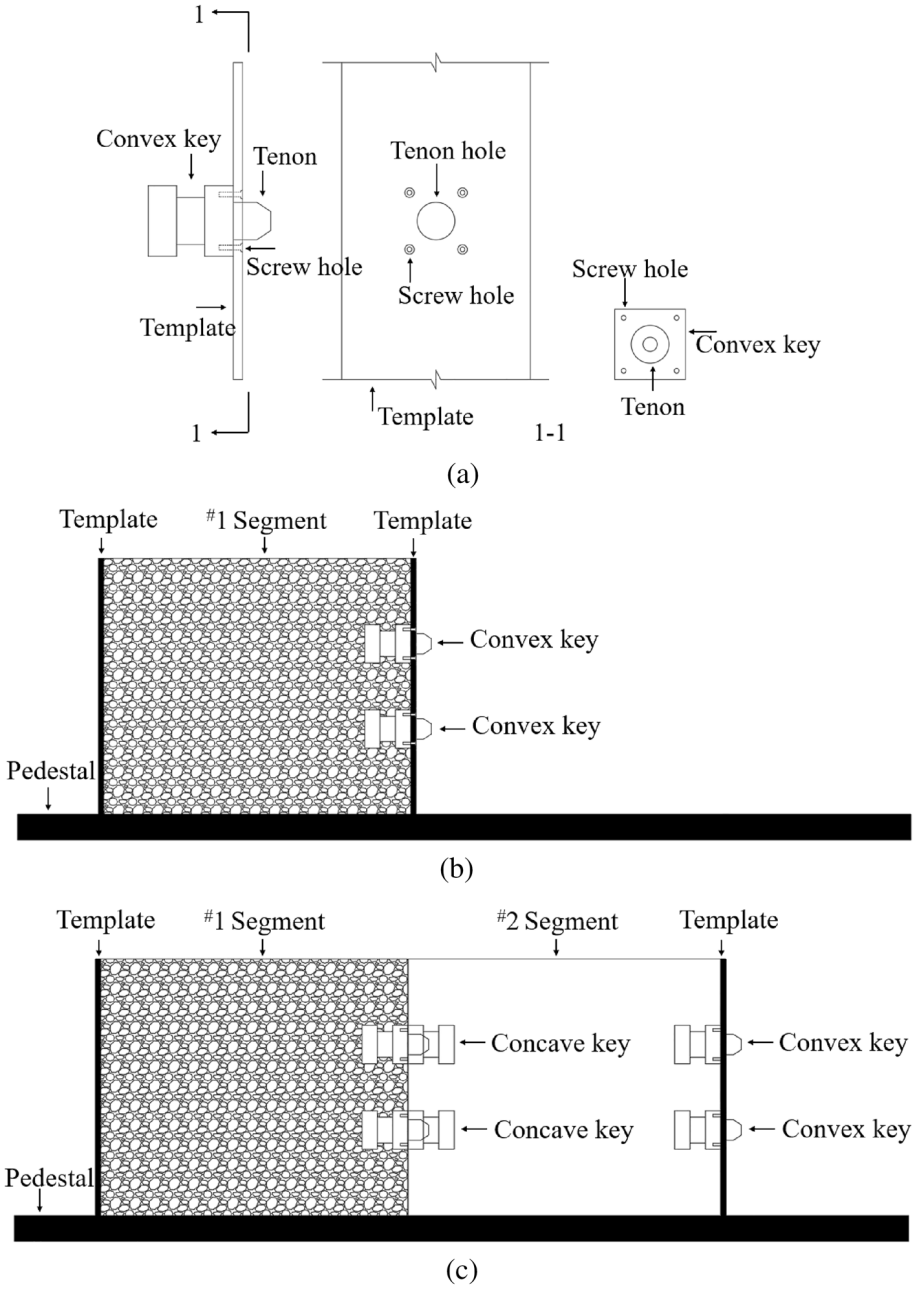
Short-line match method (**a**) template setup (**b**) preceding segment construction (**c**) sequence segment construction.

Firstly, the installation position of the steel shear key on the template was determined, holes were cut in the corresponding positions according to the diameter of the tenon and screws, and the convex key was fixed to the template with screws, as shown in Fig. 15a. Secondly, the template was installed at the planned position of ^#^1 segment, and the ^#^1 segment was poured, as shown in Fig. 15b. After the ^#^1 segment concrete reached curing age, the screws and template were removed. Thirdly, the concave keys were installed corresponding to the position of the convex keys, the template was again set up at the ^#^2 segment joint position, and the ^#^2 segment was poured, as shown in Fig. 15c. This was repeated until the construction of all segments was completed.

#### Engineering test

**Figure 16:**
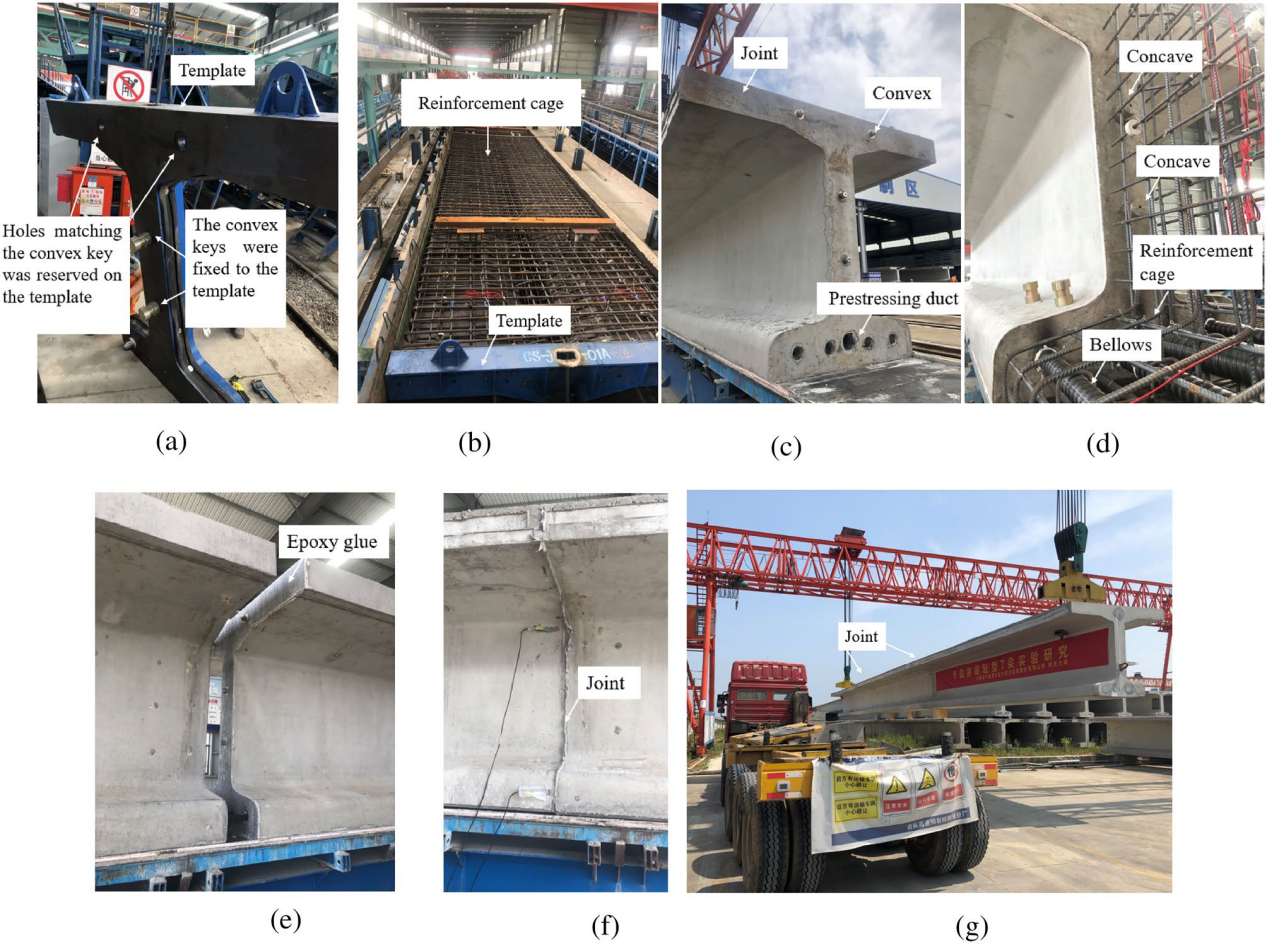
Engineering test of short-line match method(**a**) template setup (**b**) template installation (**c**) preceding segment construction (**d**) sequence segment construction (**e**) apply epoxy glue (**f**) joint assembly (**g**) hoisting.

A 3-segment 25-m T-beam was designed, and the construction method was evaluated according to the short-line match method, as shown in Fig. 16. This method uses the preceding segment as the end template of the subsequent segment, the joints are perfectly matched, the segment assembly is smooth, but the production cycle is lengthy.

### Long-line match method

#### Construction method design

**Figure 17:**
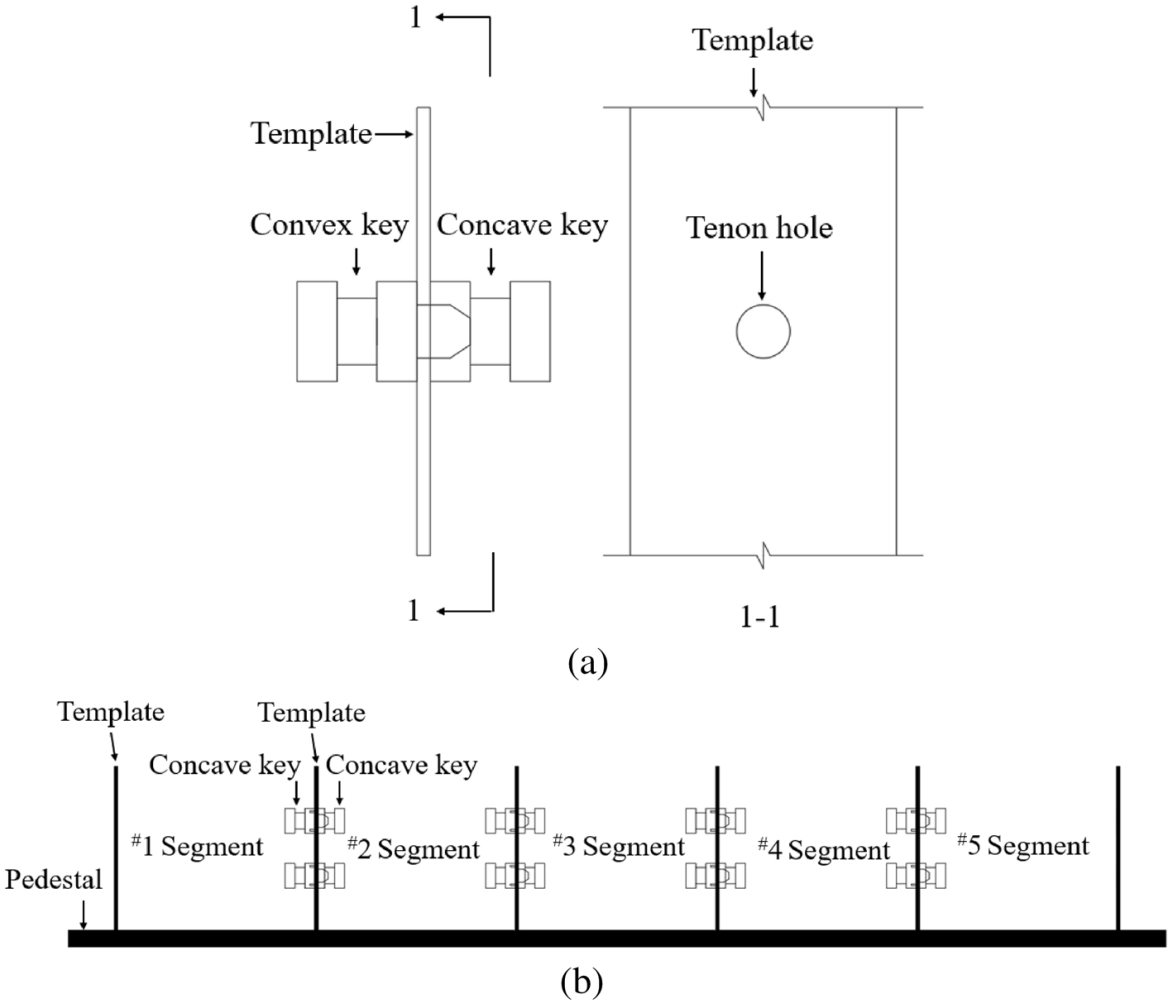
Long-line match method (**a**) template setup (**b**) multi-segment construction.

The installation position of the steel shear key on the template was determined, and holes were cut in the corresponding positions according to the diameter of the tenon, as shown in Fig. 17a. The concave and convex keys were simultaneously installed on the template, the templates were placed at the intended joint position, and the ^#^1–5 segments were then poured simultaneously, as shown in Fig. 17b.

#### Engineering test

**Figure 18:**
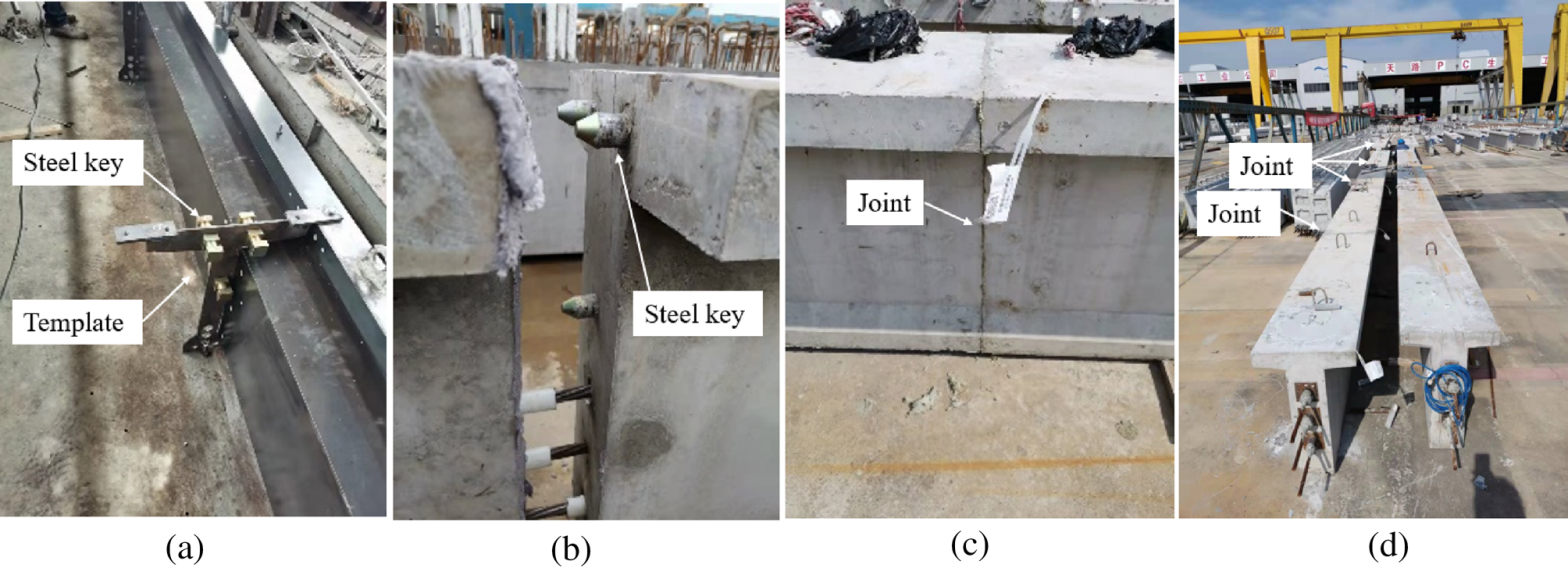
Engineering test of long-line match method (**a**) template installation (**b**) apply epoxy glue (**c**) joint assembly (**d**) assemble the parts into a whole.

A 5-segment 10-m T-beam was designed, and the construction method was designed according to the long-line match method, as shown in Fig. 18. Considering the physical deformation of the concave and convex keys caused by temperature, concrete shrinkage creep, and temporary loading, a 0.2 mm fitting tolerance was designed between the tenon and mortise. As a result, the steel key can slip during pouring. In order to solve this problem, a 0.5 mm thick rubber pad was temporarily fixed onto the tenon during the engineering test. The long-line match method greatly shortened the production cycle, but the joints did not achieve a perfect match after pouring. The flatness of the templates needed to be checked after repeated use.

### Modular construction

**Figure 19:**
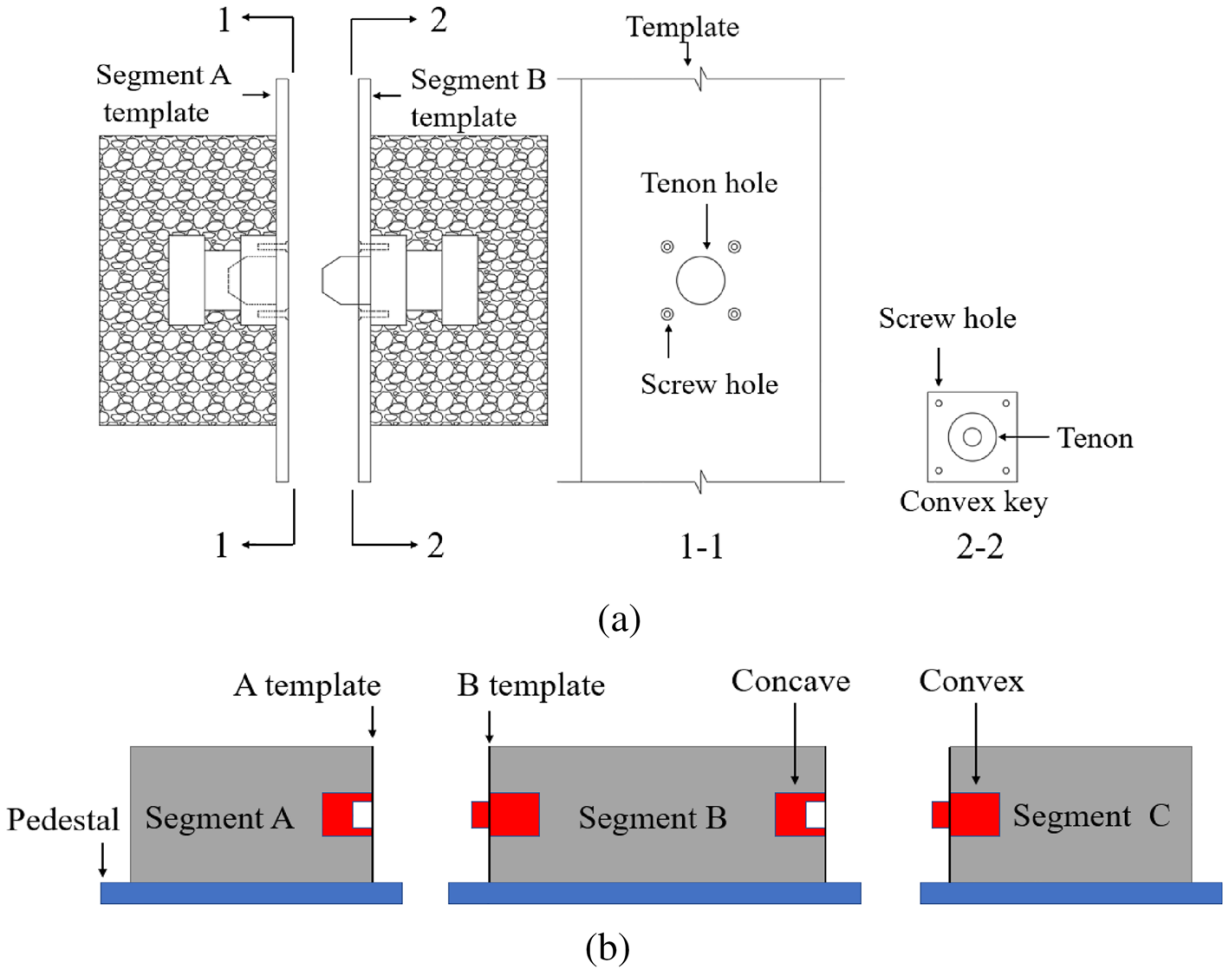
Modular construction method (**a**) template setup (**b**) independently segmental construction.

The convex and concave keys were installed in the A template and B template respectively, as shown in Fig. 19a. Segments A-C were placed on different pedestals for independent production, as shown in Fig. 19b. This method is similar to the short-line match method, but the difference is that each segment is produced independently and does not rely on the preceding segment as the end template of the sequential segment. It can greatly improve production efficiency and is especially suitable for the production of small and medium span segmental T beams and small box girders. However, the position of the concave and convex keys is completely dependent on the template, thus requiring high precision control.

## Conclusions

The steel shear keyed joint is a new type of joint in precast segment bridges, but its mechanical properties and feasible construction methods are unclear. Based on experimental research, the steel shear keyed joints have a high bearing capacity, and good ductility compared to concrete keyed joints. And based on the practical construction test, the steel shear keyed joint is suitable to standardized production. The results showed as the following:

1. Steel shear keyed joints have a high bearing capacity. Compared with concrete key joints, the bearing capacity of steel shear key dry joints is increased by 72.96% and that of epoxied joints is increased by 29.52%.
2. Steel shear keyed joints have good ductility. The bearing capacity of the concrete key joint drops suddenly following direct shear cracking. Unlike concrete key joints, the steel shear keyed joints can quickly achieve internal force redistribution to reach a new mechanical equilibrium after cracking, the joint can continue to carry the load, and the load–displacement curve exhibits a horizontal stage.
3. The structural system of steel shear keyed joints is stable after cracking. Compared with the concrete key, at the moment of cracking, the confining stress amplitude is significantly smaller, and the prestressing system is relatively more stable. After the specimen cracked, the concave and convex keys were still interlocking under the action of confining stress, and the shear resistance of the joint was still provided by the steel key. The shear slip of the steel shear key joint was significantly smaller than that of the concrete key joint, at the moment of cracking.
4. The steel shear keyed joints have factory standardized production conditions. Based on traditional concrete key construction methods and combining the advantages of a simple structure and easy installation of steel shear key, short-line match, long-line match, and modular methods were designed for steel shear keyed joint. Engineering tests were conducted with the short-line match and long-line match methods, and the test results verified that the steel shear key joints are suitable for industrial production.
5. Steel shear keyed joints have reliable mechanical properties and are suitable for industrial standard production. These have a wider application value in the field of precast segmental concrete structures such as precast segmental beams, bridge piers, cover beams, and utility tunnels.
6. Long-term exposure to air, the steel shear key will rust and their mechanical properties will decrease. Therefore, to avoid the gap in the joint surface, it is recommended that steel shear keyed joint surface coating epoxy adhesive. And we will further study the durability of the steel shear key epoxied joint.

## Data Availability

Some or all data, models, or code that support the findings of this study are available from the corresponding author upon reasonable request.

## References

[1] Zhan Y, Li Z, Chen Z, Shao J, Yue F, Liu F et al. Experimental and numerical investigations on shear performance of key tooth joints of precast concrete segmental bridge under repeated loading. Construct. Build. Mater. (2022);351.

[2] Ahmed GH, Aziz OQ (2020). Stresses, deformations and damages of various joints in precast concrete segmental box girder bridges subjected to direct shear loading. Eng. Struct..

[3] Ahmed GH, Aziz OQ (2019). Shear behavior of dry and epoxied joints in precast concrete segmental box girder bridges under direct shear loading. Eng. Struct..

[4] Hu Y, Qiu J, Li Z, Yao Y, Liu J, Wang J. Shear strength prediction method of the UHPC keyed dry joint considering the bridging effect of steel fibers. Eng. Struct. (2022); 255.

[5] Ahmed GH, Aziz OQ (2019). Shear strength of joints in precast posttensioned segmental bridges during 1959–2019, review and analysis. Structures..

[6] Jiang H, Chen L, Ma ZJ, Feng W (2015). Shear behavior of dry joints with castellated keys in precast concrete segmental bridges. J. Bridg. Eng..

[7] Jiang H, Wei R, John Ma Z, Li Y, Jing Y (2016). Shear strength of steel fiber-reinforced concrete dry joints in precast segmental bridges. J. Bridg. Eng..

[8] Jones LL (1959). Shear test on joints between precast post-tensioned units. Mag. Concr. Res..

[9] Buyukozturk O, Bakhoum MM, Michael BS (1990). Shear behavior of joints in precast concrete segmental bridges. J. Struct. Eng..

[10] Zhou X, Mickleborough N, Li Z (2005). Shear strength of joints in precast concrete segmental bridges. ACI Struct. J..

[11] Sangkhon A, Pisitpaibool C (2017). Shear strength test of joint with different geometric shapes of shear keys between segments of precast segmental bridge. Int.Trans. J. Eng. Manag. Appl.Sci. Technol..

[12] Yuan AM, Yang C, Wang JW, Chen LK, Lu RW. Shear Behavior of Epoxy Resin Joints in Precast Concrete Segmental Bridges. J. Bridge Eng.. (2019);24.

[13] Choi J-S, Lee H-J, Yuan T-F, Yoon Y-S (2023). Shear strength of steel fiber reinforced lightweight self-consolidating concrete joints under monotonic and cyclic loading. Constr. Build. Mater..

[14] Al-Rousan RZ, Qudaisat MS (2023). Single keyed joints behaviour and capacity formulation under direct shear using non-linear finite-element analysis. Structures..

[15] Zhang Y, Zhang Z, Hu F, Du X, Lu Y, Zhu J (2022). Full-scale experimental study on shear behavior of multiple-keyed epoxy joints in precast concrete segmental bridges. Structures..

[16] Alcalde M, Cifuentes H, Medina F (2013). Influence of the number of keys on the shear strength of post-tensioned dry joints. Mater. De Constr..

[17] Zhan Y, Li Z, Chen Z, Shao J, Yue F, John Ma Z (2022). Experimental and numerical investigations on shear behavior of large keyed tooth joints. Constr. Build. Mater..

[18] Luo Z, Wang Y, Wang T (2022). Shear behavior of epoxy joints in precast segmental bridges under impact loading. Eng. Struct..

[19] Freitas M, Ben Ftima M, Léger P, Bouaanani N (2022). Three-dimensional failure envelope of concrete dam shear keys. Eng. Struct..

[20] Smittakorn W, Manavithayarak P, Sukmoung P. Improvement of shear capacity for precast segmental box girder dry joints by steel fiber and glass fiber. MATEC Web of Conferences: EDP Sciences; (2019). p. 04006.

[21] Beattie SM. Behavioral improvements in segmental concrete bridge joints through the use of steel fibers. Massachusetts: Massachusetts Institute of Technology; (1989).

[22] Park S-H, Dinh NH, Kim S-H, Lee S-J, Choi K-K (2021). Direct shear behavior of precast panel connections with cast-in-place shear keys using steel fiber-reinforced cementitious mortar (SFRCM). Structures..

[23] Voo YL, Foster SJ, Voo CC (2015). Ultrahigh-performance concrete segmental bridge technology: Toward sustainable bridge construction. J. Bridg. Eng..

[24] Gopal BA, Hejazi F, Hafezolghorani M, Lei VY (2019). Numerical analysis and experimental testing of ultra-high performance fibre reinforced concrete keyed dry and epoxy joints in precast segmental bridge girders. Int. J. Adv. Struct. Eng..

[25] Kim YJ, Chin WJ, Jeon SJ (2018). Interface shear strength at joints of ultra-high performance concrete structures. Int. J. Con. Struct. Mater..

[26] Sun X (2015). Experimental study on shear behavior of joints in precast segmental bridges.

[27] Issa MA, Abdalla HA (2007). Structural behavior of single key joints in precast concrete segmental bridges. J. Bridg. Eng..

[28] Rombach G (2002). Precast segmental box girder bridges with external prestressing-design and construction.

[29] Turmo J, Ramos G, Aparicio A (2006). Shear strength of dry joints of concrete panels with and without steel fibres: Application to precast segmental bridges. Eng. Struct..

[30] Shamass R, Zhou X, Alfano G (2015). Finite-element analysis of shear-off failure of keyed dry joints in precast concrete segmental bridges. J. Bridg. Eng..

[31] Zou Y, Xu D (2022). Experimental study on shear behavior of joints in precast concrete segmental bridges. Structures..

[32] China IsotPsRo. Specification for design of highway reinforced concrete and prestressed concrete bridges and culverts (JTG 3362–2018). Beijing: China Communications Press.

[33] Aashto IJS. Guide specifications for design and construction of segmental concrete bridges. (1999).

[34] Roberts CL, Breen JE, Kreger ME. Measurement based revisions for segmental bridge design and construction criteria: Center for transportation research. Bureau of Eng. Res. (1993).

[35] Zou Y;Xu D. Shear behavior of steel keyed joints in precast concrete segmental bridges under direct shear loading. Struct. Con. (2022).

[36] Yuan Aimin FJ, Cheng Leike, et al. Experiment of shear performance of epoxy resin joints with reinforced keys in precast concrete segmental bridge. Chin. J. Highway Trans. (2018); 31:85–91.

[37] Zou Y, Xu D (2021). Mechanical characteristics of steel shear keyed joints in the construction and finished states. Adv. Civil Eng..

